# Isolation of Polygalacturonase-Producing Bacterial Strain from Tomatoes (*Lycopersicon esculentum* Mill.)

**DOI:** 10.1155/2019/7505606

**Published:** 2019-01-15

**Authors:** Yemisi Dorcas Obafemi, Adesola Adetutu Ajayi, Olugbenga Samson Taiwo, Shade John Olorunsola, Patrick Omoregie Isibor

**Affiliations:** ^1^Department of Biological Sciences, Covenant University, PMB 1023, Ota, Ogun State, Nigeria; ^2^Department of Biological Sciences, Augustine University, PMB 1010, Epe, Lagos State, Nigeria

## Abstract

**Background:**

Polygalacturonase (EC 3.2.1.15) enzyme aids in microbial spoilage of fruits and vegetables. It is very important to find economical ways to producing the enzyme so as to achieve maximum yield in industries due to its use at different areas of production process.

**Methods:**

Isolation of polygalacturonase-producing bacterial strain from tomatoes (*Lycopersicon esculentum* Mill.) was studied. Polygalacturonase-producing bacterial strains were isolated and screened from tomatoes stored at normal laboratory temperature (25 ± 2°C). They were identified based on their morphological, biochemical, and molecular characteristics. The enzyme produced was partially purified by the ammonium sulphate precipitation method. Molecular weights and optimum conditions for best enzyme activity were obtained by SDS PAGE technique.

**Results:**

Five bacterial isolates resulted after screening. Bacterial strain code B5 showed highest polygalacturonase activity. Optimum conditions for polygalacturonase PEC B5 were maintained at pH 4.5; temperature 35°C; substrate concentration 0.3 mg/ml, and best activity at less than 5 min of heating. The enzyme PEC B5 was found to weigh 65 kDa and 50 kDa for crude and partially purified aliquots, respectively. The result of 16S rRNA gene sequencing revealed bacterial strain code B5 as *Enterobacter tabaci NR146667* having 79% similarity with the NCBI GenBank.

**Conclusion:**

Microorganisms should be developed for large-scale production of enzymes in developing countries.

## 1. Introduction

Microbial enzymes play an important role in catalyzing several metabolic reactions [1, 2]. There is high demand for enzymes in the industries as there is continuous need for sustainable development [3]. Microorganisms serve as one of the largest and useful sources of many enzymes [4]. Microorganisms can produce different cell wall degrading enzymes which breakdown large polysaccharides into simple reducing sugars that are used up for growth and multiplication of microorganisms [5]. These enzymes have a lot of industrial and environmental importance [2, 6]. They are usually used in the brewery, food processing, and bioremediation of organic pollutants [7, 8].

Tomato (*Lycopersicon esculentum* Mill.) fruits are important sources of our nutrients as they supply the body with the necessary food components which are vital for our growth and development [9]. However, they become deteriorated during their harvest, transportation or storage due to the climatic, microbial growth, and pest attack on their cell walls [10]. Over a long period of time, there has been an urgent need to isolate and identify microorganisms that are found to often cause spoilage of fruits, as well as to characterize the extracellular enzymes produced by the microorganisms in order to find a way to preserve the tomato fruits all year round and produce enzymes for industrial use [11]. Many pathogenic bacteria and fungi are known to produce some extracellular enzymes which can degrade the cell wall of the tomato fruits [12, 13]. The enzymes are involved in tissue breakdown of the fruits, and this play a major role in helping the microorganism to penetrate the cell wall of the plant [1, 14].

Tomato fruits are highly susceptible to microbial attack due to large variance in their chemical components such as pH and moisture content which makes them more susceptible to microbial proliferation within a short period of time [11].

The primary cell wall components of all plants are pectins [15]. The polysaccharides which are rich in polygalacturonic acid have two mains regions which are the smooth and hairy regions [16]. Polygalacturonase enzyme (EC 3.2.1.15) plays a very crucial role in opening of the plant cell wall, thereby causing infection in the host plants [17]. The fact that enzymes such as polygalacturonase play important roles in deterioration of important fruits have been previously reported [9]. The major environmental factors affecting polygalacturonase activities are pH and temperature [2, 18]. The objective of our study is to develop isolate bacterial strains that could produce polygalacturonase enzyme from tomato fruits.

## 2. Materials and Methods

### 2.1. Collection of Samples

Two-hundred fresh tomato fruits were purchased from Iju market, in Ado-Odo Local Government Area in Ogun State, Nigeria. The tomatoes were sorted, washed, and transported to the Microbiology Laboratory in Covenant University, Ota, Ogun State in sterile polythene bags in August, 2017. A set of hundred fruits designated AT were stored at ambient temperature while the second set of hundred fruits designated CT were stored in the refrigerator as the control. All samples were observed, and sampling was done for a period of fifteen days.

### 2.2. Isolation of Bacterial Strains from Tomato Fruits

The total bacterial population was determined using the pour plate method; each sample of the tomato fruit was diluted with sterile distilled water. One milliliter of the dilution of 10^4^ was plated onto Nutrient Agar (NA) incubated at 37°C for 24 h.

### 2.3. Bacterial Strain Identification

Selected bacterial colonies were identified using basic microbiology morphological and biochemical techniques with the help of taxonomic scheme of Bergey's Manual of Determinative Bacteriology [19].

### 2.4. Selection of Polygalacturonase-Producing Bacteria

The screening was done according to the methods described [20] Polygalacturonase supplemented agar (PSA) is used for the selective growth of those microorganisms which releases pectin. The medium was prepared using the following chemicals: 0.3 g/100 ml of (NH_4_)_2_HPO_4_, 0.2 g/100 ml of KH_2_PO_4_, 0.3 g/100 ml of K_2_HPO_4_, 0.01 g/100 ml of MgSO_4_, 2.5 g/100 ml agar, 1 g/100 ml of powdered pectin, and pH 4.5. The PSA plates were inoculated using a circular streak on the agar and incubated at 37°C for 4 days. Growth on PSA plates were observed by measuring the diameter of hydrolysis area produced by the bacterial strains on the agar plates [20].

### 2.5. Preparation of Standard Bacterial Inoculum for Production of Enzyme

The bacterial colonies with highest zone of hydrolysis was subcultured into 25 ml Lactose Broth and incubated at 37°C for 24 h [18].

### 2.6. Extraction of Polygalacturonase Enzyme

The mixture was filtered [21] using muslin cloth, Whatman No. 1 filter paper. The resulting filtrate is then centrifuged at 5,000 rpm for 30 minutes. The filtrate is the crude enzyme while the bacterial cells residue was discarded [21].

### 2.7. Polygalacturonase Assay

Polygalacturonase enzyme activity was obtained by calculating volume of reducing sugar produces in medium which contained 1 ml of 0.1% (w/v) pectin (Sigma) in 0.1 M citrate phosphate buffer pH 4.5 and 0.5 ml of crude enzyme boiled at 100°C for 15 minutes. The experimental and control medium were incubated at 35°C for 3 hours. Termination of reaction was done by adding 3 ml of 3,5-dinitrosalisylic acid (DNSA) reagent [22], and 1 ml of crude enzyme was added to each control medium. The medium was heated at 100°C in boiling water for 15 min and cooled to room temperature. Optical density was taken at absorbance reading at 540 nm. Released reducing sugars were estimated by the modified dinitrosalicylic acid reagent as described [22].

### 2.8. Ammonium Sulphate Precipitation

Ammonium sulphate of analytical grade was added to crude enzyme preparation to 90% saturation [9, 20]. The solution was kept for 24 h at 4°C. Removal of precipitate was done by centrifuging at 5000 rpm for 30 minutes, the supernatant was decanted, and the precipitate was then dissolved again in 1 ml of 0.1 M citrate phosphate buffer (pH 4.5) [4, 23]. Dialysis of the enzyme was done in acetylated cellophane tubing prepared from Visking dialysis tubing (Gallenkamp) [24]. The protein content of the dialysate was determined [25]. The enzyme assay of the content of the dialysate was also determined as described [22].

### 2.9. Effects of Temperature on the Polygalacturonase PEC B5

The substrate was 0.1% (w/v) pectin in 0.1 M citrate phosphate buffer (pH 4.5). The medium containing 1 ml of substrate and 0.5 ml of polygalacturonase were incubated at different temperatures of 20°C–45°C. After 3 hours of incubation at each temperature, polygalacturonase activity was determined [22].

### 2.10. Effects of pH on Polygalacturonase PEC B5

The substrate was 0.1% (w/v) pectin in 0.1 M citrate phosphate buffer of varying pH values ranging from pH 2.0–4.5. The medium containing 1 ml of pectin and 0.5 ml of polygalacturonase was incubated at 35°C for 3 h, and the activity was determined [22].

### 2.11. Effects of Substrate Concentrations on Polygalacturonase PEC B5

The effect of various substrate concentrations was determined using different concentrations of pectin in 0.1 M citrate phosphate buffer; 0.05%–0.25%. The reaction mixture contained 1 ml of pectin, and 0.5 ml of polygalacturonase was incubated at 35°C for 3 h. Polygalacturonase activity was determined [22].

### 2.12. Effect of Heating Time (80°C) on Stability of Polygalacturonase PEC B5

The effect of time of heating on stability of polygalacturonase enzyme was determined. Samples of partially purified enzymes were heated at 80°C for different periods of time (0–30 minutes), respectively. The medium containing 1 ml of pectin and 0.5 ml of polygalacturonase was incubated at 35°C for 3 h, and polygalacturonase activity was determined [22].

### 2.13. Extraction and Characterization of Deoxyribonucleic Acid (DNA) from Isolated Bacterial Species

The deoxyribonucleic acid (DNA) extraction was carried out on the bacteria samples using the Jena Bioscience Bacteria DNA Preparation Kit purchased from Jena Bioscience JmbH, Jena, Germany. The DNA was amplified using the primer pair 27F-5′-AGAGTTTGATCCTGGCTCAG-3′ and 1492R-5′-GGTTACCTTGTTACGACTT-3′ by polymerase chain reaction technique. Molecular characterization of polygalacturonase-producing bacterial strain was done by 16S rRNA gene sequencing technique. All PCR products were purified and sent to Inqaba (South Africa) for Sanger sequencing, and the corresponding sequence was identified using the National Center for Biotechnology Information (NCBI) blast.

### 2.14. Molecular Weight Determination of Polygalacturonase PEC B5

The molecular weight of the enzyme was determined by comparing them with the molecular weight marker [18], and ammonium sulphate precipitated sample was run on Sodium dodecyl sulphate polyacrylamide gel electrophoresis (SDS PAGE). The native gel placed over the pectin agar gel was then subjected to zymogram staining [26].

## 3. Results

### 3.1. Enumeration of Bacterial Population

**Figure 1:**
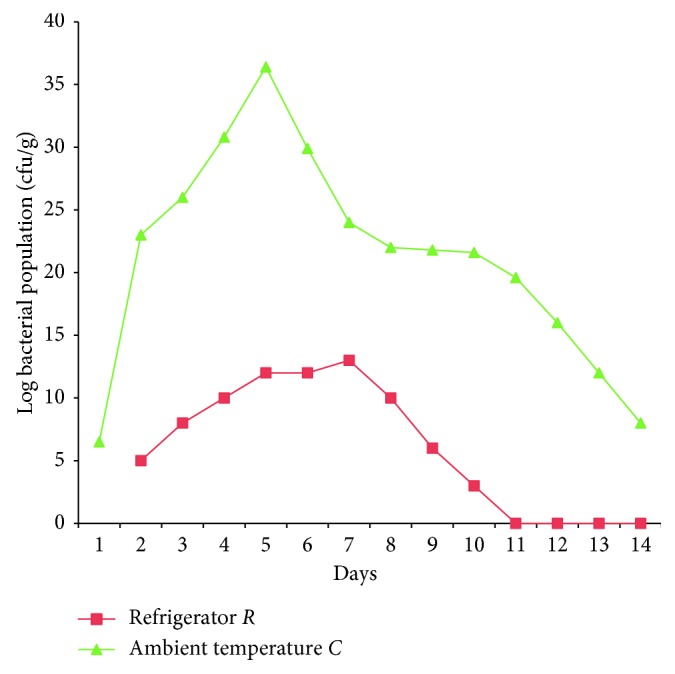
Enumeration of bacterial population.

The total bacterial count for the tomato fruits stored in the refrigerator had bacterial count range of 1.0 × 10^4^ – 3.0 × 10^6^ cfu/g and 2.0 × 10^4^ – 2.0 × 10^14^ cfu/g from the fruits stored at ambient temperature (Figure 1).

### 3.2. Identification of Bacterial Isolates

Pure bacterial isolates were identified as *Aeromonas* sp., *Bacillus* sp., *Citrobacter* sp., *Klebsiella* sp., *Enterobacter* sp., *Staphylococcus* sp., *Lactobacillus* sp., and *Micrococcus* sp. (Table 1).

### 3.3. Screening of the Pectinolytic Bacterial Isolates

**Figure 2:**
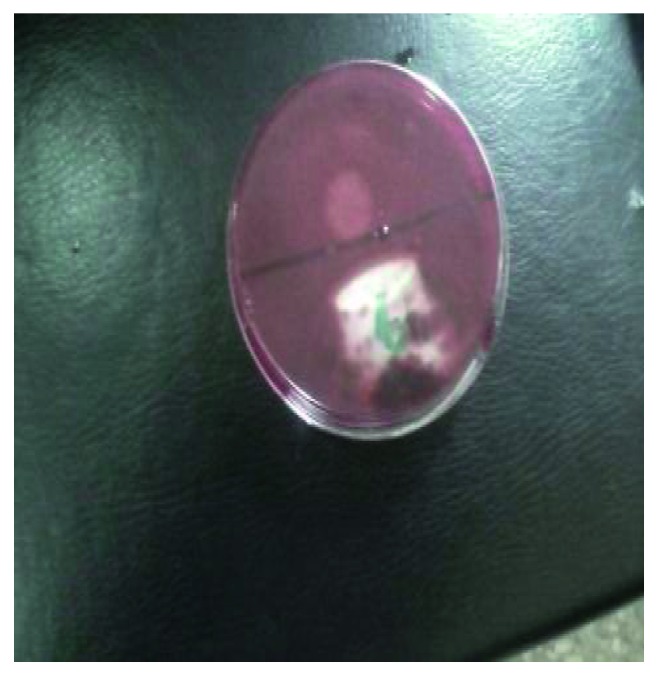
Zone of hydrolysis on pectin agar.

Bacterial isolates obtained from tomato stored at room temperature and in the refrigerator were screened for polygalacturonase production (Figure 2). Isolate code B5 was selected for further studies based on the ability to produce larger zone of hydrolysis measuring 60 mm (Table 2).

### 3.4. Production of Polygalacturonase

The polygalacturonase produced by bacterial isolate code B5 isolated from fruits kept at room temperature on day 5 had a total activity of 0.911 units/ml, protein content of 0.600 mg/ml, a specific activity of 1.518 units/mg protein, and 72% yield after ammonium sulphate precipitation (Table 3).

### 3.5. Effect of Temperature on Polygalacturonase PEC B5

**Figure 3:**
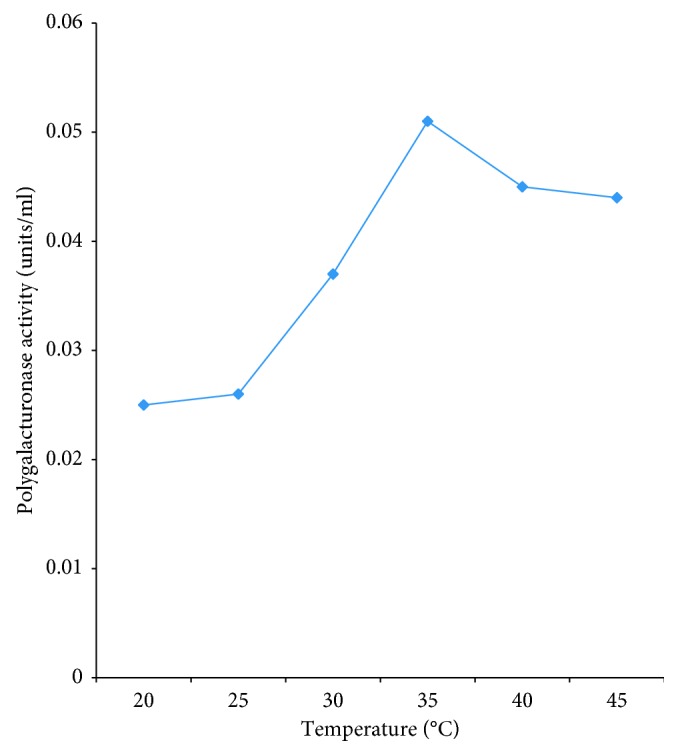
Effect of temperature on partially purified polygalacturonase PEC B5.

The temperature of incubation affected polygalacturonase activity tremendously increasing the activity of PEC B5 with an increase in incubation temperature (Figure 3). Further increase beyond the optimum temperature reduced the enzyme activity. The optimum temperature for PEC B5 was 35°C.

### 3.6. Effect of pH on Polygalacturonase PEC B5

**Figure 4:**
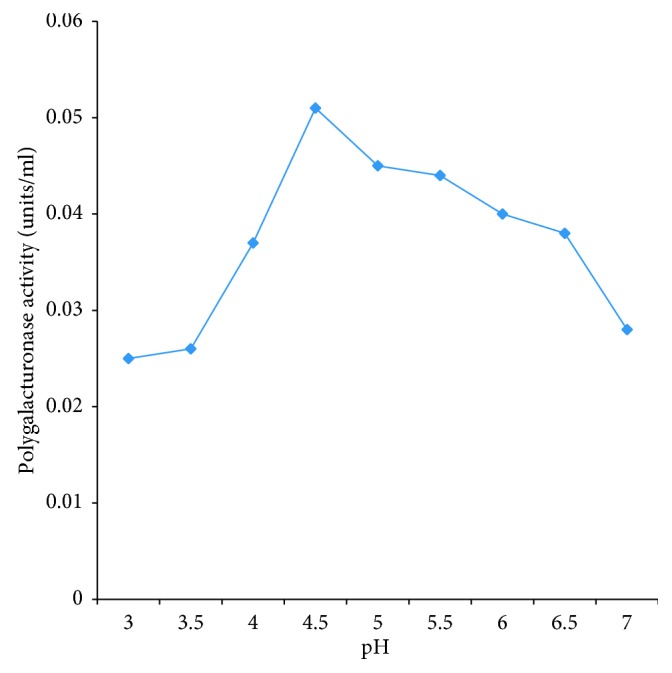
Effect of pH on partially purified polygalacturonase PEC B5.

The enzyme activity increased as the pH increases and also decreased when the optimum pH value was reached. The optimum pH for polygalacturonase activity for PEC B5 was 4.5 (Figure 4).

### 3.7. Effect of Substrate Concentration on Polygalacturonase PEC B5

**Figure 5:**
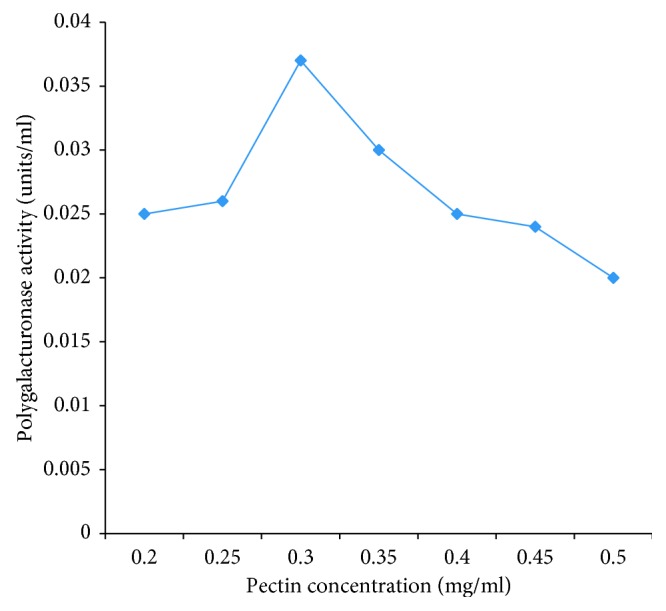
Effect of pectin concentration on partially purified polygalacturonase PEC B5.

The activity of the polygalacturonase produced increases with an increase in the concentration of the substrate. This continued to increase until an optimum concentration of substrate was attained (Figure 5). The optimum substrate concentration of substrate of PEC B5 was 0.3 mg/ml.

### 3.8. Effect of Heat (80°C) on Polygalacturonase PEC B5

**Figure 6:**
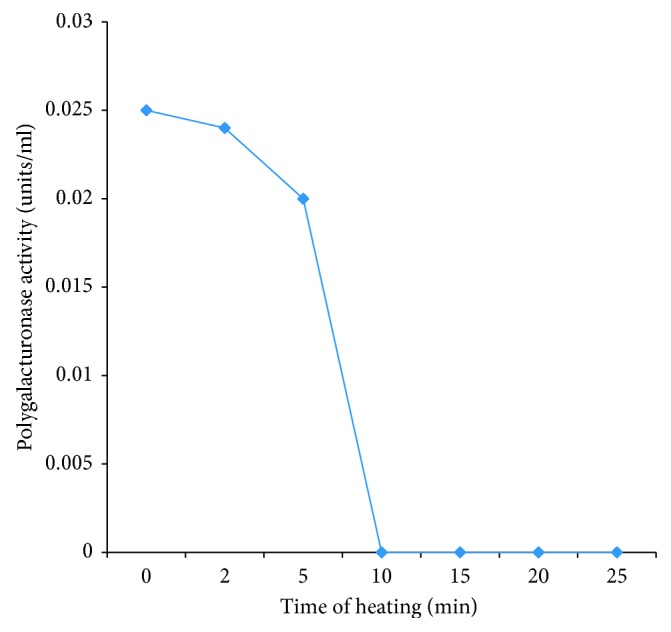
Effect of heat (80°C) on partially purified polygalacturonase PEC B5.

The activity of polygalacturonase on heating at 80°C decreases with longer time of heat application. When polygalacturonase was subjected to heat for 5 minutes, activities of polygalacturonase was optimum. The polygalacturonase activity was completely inactivated after 10 minutes of heating (Figure 6).

### 3.9. Polymerase Chain Reaction (PCR) Amplification and Molecular Characterization of Bacterial Isolates Using the 27F and 1492R Primer Pair

**Figure 7:**
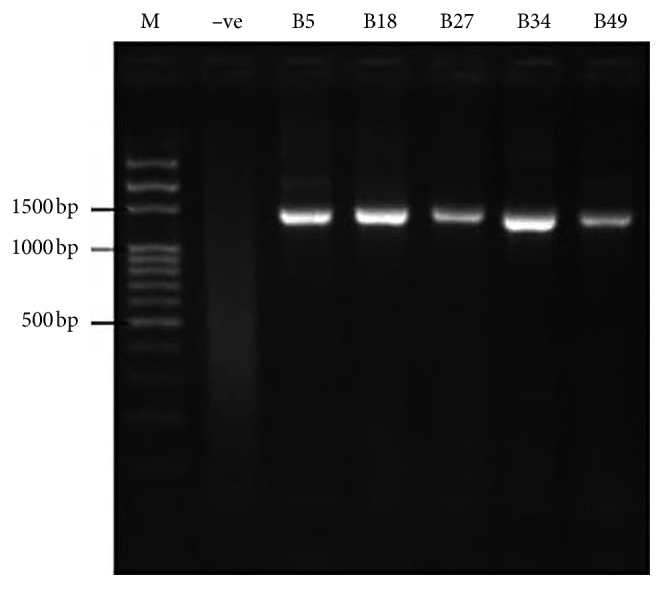
Polymerase chain reaction electropheretic gel of isolated bacterial strains using 27F and 1492R primer pair. M: 500 bp DNA ladder. −ve: bacterial control. B5: isolate code B5. B18: isolate code B18. B27: isolate code B27. B34: isolate code B34. B49: isolate code B49.

Bacterial isolates were characterized by the presence of a constant 1500 bp amplicon size. The negative control does not have this size (Figure 7). Hence, B5, B18, B27, B34, and B49 are all bacterial isolates. The result of 16S rRNA gene sequencing identified isolate B5 as *Enterobacter tabaci* NR146667 with 79% matched with NCBI-blasted record. The other bacterial isolates B18 and B27 were identified as *Enterobacter aerogenes* LT703513.1 with 98% similarity and *Citrobacter freundii* KU570343.1 with 99% similarity when matched with NCBI-blasted record. The last two isolates B34 and B49 could not be identified because their sequences were too short for query.

### 3.10. Molecular Weight of Polygalacturonase PEC B5

**Figure 8:**
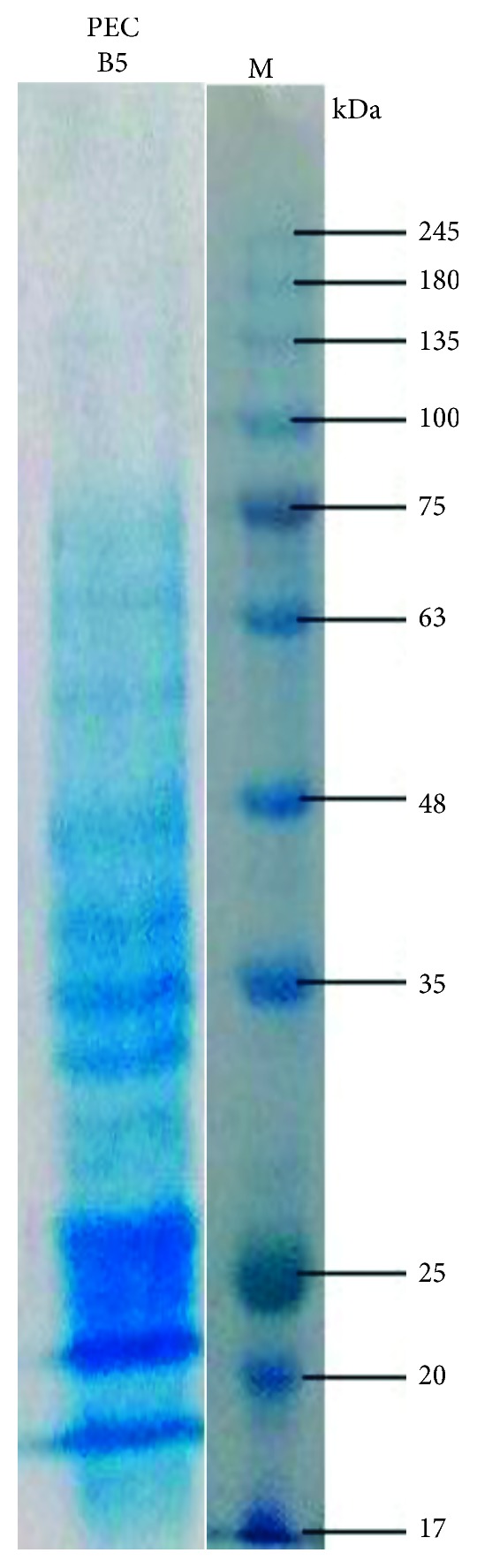
The SDS PAGE of the crude polygalacturonase PEC B5 enzyme.

**Figure 9:**
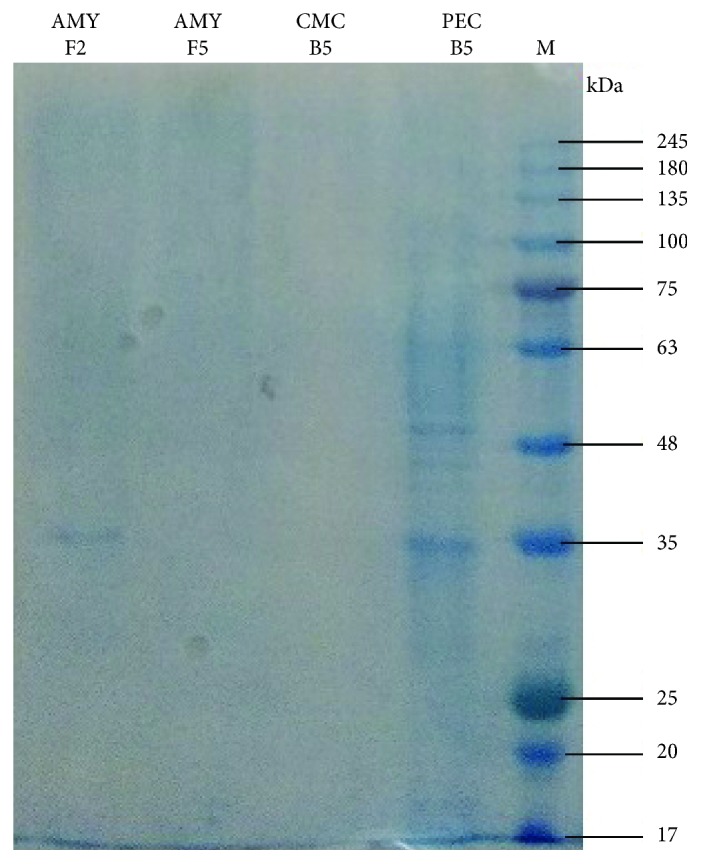
The SDS PAGE of the partially purified polygalacturonase PEC B5 enzyme.

The molecular weight of extracted crude polygalacturonase PEC B5 and partially purified polygalacturonase PEC B5 are 65 kDa and 50 kDa, respectively (Figures 8 and 9).

## 4. Discussion

This study revealed capability of some bacterial strains to produce extracellular enzymes such as the polygalacturonase (EC 3.2.1.15). Production of polygalacturonase from fresh tomato fruits was reported [2, 4, 27]. Microbial production of polygalacturonase from bacteria, fungi, and yeasts has also been reported [28]. Extraction of enzymes from microorganisms was also isolated from other sources such as from other fruits and vegetables, bread, Irish potato, and even soil were also reported [13, 15, 29–32].

Morphological, biochemical, and 16S rRNA gene sequencing results identified polygalacturonase-producing bacterial isolates as members of the genera *Enterobacter*, *Klebsiella, Pseudomonas, Escherichia*, and *Citrobacter*. The result is similar to the previous report [4]. High prevalence of members of the *Enterobacteriaceae* on the tomato fruits indicates their possible potential to cause food-borne illness and contamination which may be due to postharvest handling [33]. The optimum temperature of the polygalacturonase produced in this study was 35°C. This is in support of previous report [34]. However, this report contrasts with earlier reports [35, 36] which implied that polygalacturonase production by some bacterial strains was optimum at 37°C. Optimum pH was identified as 4.5 for polygalacturonase PEC B5. This result conforms with a report that polygalacturonase was most active at pH 5.0 [37]. The optimum substrate concentration for the pectin was 0.3 mg/ml. Previous research [38] also reported optimum substrate concentration 0.25 mg/ml for polygalacturonase enzyme. The effect of heating time which was investigated at 80°C over a period of 30 min revealed loss of enzyme activity within 10 min of heating. This confirmed earlier reports of various researchers [9, 37, 38]. The report [37] revealed that polygalacturonase enzyme was active for over 5 min of heating at 80°C. There was also a continuous reduction in the enzyme activity as long as heat was applied. After 10 min of heating, enzyme activity was completely lost. The analysis [23, 37] also reported that polygalacturonase enzyme lost 10% of their activity at 70°C [18].

Many researchers and analysts have implicated polygalacturonase and other pectin related enzymes in pathogenicity [15, 39, 40].

## 5. Conclusion

The research discovered important bacterial strains isolated from tomato (*Lycopersicon esculentum* Mill.) fruits which have polygalacturonase enzyme-producing capability. This property will make it very helpful in producing important enzymes in food, drinks, and pharmaceutical industries at affordable prices.

## Figures and Tables

**Table 1:** Identification of bacterial isolates.

Isolate code	Morphology	Gram staining reaction	Catalase	Oxidase	Coagulase	Citrate	Urease	MR-VP	Motility	Lactose	Glucose	Sucrose	Bacteria isolates
B5	Cream round raised	Gram-negative rods	+	+	−	+	+	−	−	NR	AG	AG	*Klebsiella* sp.
B18	Cream round raised	Gram-negative rods	+	−	−	+	+	+	+	NR	AG	AN	*Enterobacter* sp.
B27	Cream round raised	Gram-negative rods	+	−	−	+	+	+	+	NR	AG	AG	*Citrobacter* sp.
B34	Cream round raised	Gram-positive cocci	+	−	−	−	+	+	N	NR	AG	AN	*Micrococcus* sp.
B49	Cream round flat	Gram-negative rods	+	−	−	+	−	−	+	AN	AG	AN	*Aeromonas* sp.

+, positive; −, negative; Ab, absent; Pr, present; AN, acid no gas; AG, acid and gas production; NR: no reaction.

**Table 2:** Zones of hydrolysis of pectinolytic bacteria.

Isolate code	Diameter of zone of hydrolysis on pectin agar (mm)
B5	60
B18	15
B27	0
B34	0
B49	32

**Table 3:** Partial purification of polygalacturonase obtained from bacterial isolate B5.

Enzyme code	Protein step	Total activity (units/ml)	Protein (mg/ml)	Specific activity (units/mg)	Yield (%)	Purification fold
PEC B5	Crude enzyme	0.91	0.60	1.52	100	1.00
	Ammonium sulphate precipitation	0.66	0.03	24.30	72	1.60

## Data Availability

The data used to support the findings of this study are available from the corresponding author upon request.
